# Responses of root characteristics and nitrogen absorption and assimilation to different pH gradients of winter wheat at seedling stage

**DOI:** 10.1371/journal.pone.0293471

**Published:** 2023-12-21

**Authors:** Chenchen Shi, Peiyu Wang, Guangtao Wang, Tiezhu Hu, Zhengang Ru, Suwei Feng

**Affiliations:** Henan Provincial Key Laboratory of Hybrid Wheat, Henan Institute of Science and Technology / Collaborative Innovation Center of Modern Biological Breeding, Xinxiang, China; Tennessee State University, UNITED STATES

## Abstract

Nitrogen (N) and rhizosphere pH are the two main factors restricting the growth of winter wheat (*Triticum aestivum* L.) in North China Plain. Soil nutrient availability is affected by soil acidity and alkalinity. In order to understand the effect of rhizosphere pH value on wheat nitrogen metabolism and the response of wheat growth to pH value at seedling stage, winter wheat varieties ‘Aikang 58’ (AK58) and ‘Bainong 4199’ (BN4199) were tested in hydroponics under three pH treatments (pH = 4.0, 6.5, and 9.0). The results showed that the accumulation of dry matter in root and above ground under pH 4.0 and pH 9.0 treatments was lower than that under pH 6.5 treatments, and the root/shoot ratio increased with the increase of pH value. Regardless of pH value, ‘BN4199’ had higher root dry weight, root length, root surface area, root activity and root tip than ‘AK58’. Therefore, wheat that is tolerant to extreme pH is able to adapt to the acid-base environment by changing root characteristics. At pH 4.0, the net H^+^ outflow rate of wheat roots was significantly lower than that of the control group, and the net NO_3_^−^ flux of wheat roots was also low. The net H^+^ outflow occurred at pH 6.5 and 9.0, and at the same time, the net NO_3_^−^ flux of roots also increased, and both increased with the increase of pH. The activity of nitrate reductase (NR) in stem of pH 9.0 treatment was significantly higher than that of other treatments, while the activity of glutamine synthetase (GS) in root and stem of pH 6.5 treatment was significantly higher than that of other treatments. Under pH 4.0 and pH 9.0 treatments, the activities of NR and GS in ‘BN4199’ were higher than those in ‘AK58’, The root respiration of ‘BN4199’ was significantly higher than that of ‘AK58’ under pH 4.0 and pH 9.0 treatment, and ‘BN4199’ had higher NO_3_^−^ net flux, key enzyme activity of root nitrogen metabolism and root respiration. Therefore, we believe that ‘BN4199’ has strong resistance ability to extreme pH stress, and high root/shoot ratio and strong root respiration can be used as important indicators for wheat variety screening adapted to the alkaline environment at the seedling stage.

## Introduction

Winter wheat (*Triticum aestivum* L.) is a major crop in China and plays an important role in national food security. However, the grain yield and quality of wheat were significantly affected by soil pH. The acidity and alkalinity of soil is important chemical properties of soil, which has a profound impact on the growth of microorganisms and crops, as well as the physical properties of the soil and the availability of nutrients [1, 2], thereby affecting soil fertility and plant growth and development, and limiting crop planting scope. Acidic and alkaline soils are widely distributed in China, among which nearly 150 million mu of saline-alkali land has potential for development and utilization, while acidic soils are mainly distributed in the subtropical south and tropical areas, with an area of 2.18 × 10^6^ km^2^, accounting for 22.7% of the total land area of the country [3]. The conservation of these lands serves as a strategic reserve to safeguard the security of national arable land, while their sustainable development and utilization form the fundamental basis for ensuring national food security.

The pH of soil environment has a great influence on the availability of soil nutrients, and most nutrients are more effective when neutral (6.5–7.5) [4]. Abnormal pH environment in soil will affect the further improvement of wheat yield [5], and soil pH value is greatly affected by the concentration of H^+^ in soil environment. In wheat production, the root system is in direct contact with the soil environment and is an important organ for absorbing and transporting water and nutrients [6]. Hinsinger et al. [7] suggested that root-induced proton outflow or inflow can lead to a significant decrease or increase in rhizosphere pH, respectively, usually up to ± 0.1 to 1 pH unit, and in some cases up to ± 2–3 pH units. Adding acidic or alkaline chemicals to soil improvement can cause changes in soil physical, chemical and biological processes, which are closely related to the effectiveness of soil nutrients and the production of harmful substances, thus affecting soil fertility and indirectly affecting plant growth and development [8, 9]. However, it is still unknown how the application of acidic or alkaline chemicals will respond to plants and improve the environment, which is influenced by various local conditions [10, 11]. But at the same time, acidic or alkaline chemical materials are not readily available in many areas, the cost of large area application can be prohibitive, and subsoil acidity and alkalinization cannot be corrected by surface application of soil amendments. Therefore, the current mainstream direction of agricultural production lies in the selection of wheat varieties that exhibit tolerance towards abnormal soil pH levels. Borzouei et al. [12] found that wheat varieties with strong tolerance to acidic or alkaline soil had better growth characteristics, and the yield of tolerant wheat varieties was higher than that of sensitive varieties [13]. This is because there are great differences in acid and alkali resistance of different varieties [14, 15]. Therefore, it is of great significance to select wheat varieties that can adapt to soil pH changes for stress tolerance breeding.

The effects of soil acidity and alkalinity on crop growth are mainly due to nutrient absorption limitations and the presence of high levels of toxicity to plants [16, 17]. Compared with neutral pH, higher soil pH increases the resistance of plant root growth, weakens the ability of roots to absorb ions, and disturbs the ion balance of plant rhizosphere [18, 19]. Rhizosphere pH affects root uptake of nitrate [20]. Liu et al. [21] found that the rhizosphere environment of wheat affected the change of net H^+^ flux in roots, while most nutrient transport in plant roots was coupled with and driven by H^+^ gradient [22, 23]. Nitrogen is the nutrient absorbed by most plants at the highest rate, and the nitrate transporter encoding the plasma membrane H^+^/NO_3_^−^ transporter promotes nitrate absorption [24]. Therefore, it plays a prominent role in cation-anionic equilibrium [25, 26]. Since crop roots can neutralize the acidity of rhizosphere soil by absorbing NO_3_^−^ or releasing OH^−^ Australian scholars have established a biological improvement method for acidic soil [4] to manage the absorption of nitrate for biological improvement to combat the acidity of subsoil in agricultural systems. Studies have shown that silicon can enhance nitrogen assimilation and chlorophyll synthesis in cucumber, especially NO_3_^−^ absorption by root system [27]. The ability of wheat to absorb and assimilate nitrates has a direct effect on grain quality [28]. The application of nitrates and phosphates locally has been demonstrated to enhance root proliferation in wheat and optimize rhizosphere alkalization in acidic subsoils [29]. Fageria and Baligar [30] concluded that crops respond differently to soil pH, wheat has the worst acid tolerance, and most trace element concentrations increase with increasing soil pH. Therefore, soil pH value affects soil nutrient availability, which in turn affects fertilization effect and fertilizer utilization efficiency.

Since the root system is the first plant site to respond to stress, it is highly relevant to use the root system to study the effects of different pH levels on wheat growth. Previous studies have shown that rhizosphere pH has significant effects on plant growth, root morphology and nitrogen use efficiency [28, 29, 31]. However, the effects of rhizosphere pH environment on nitrogen uptake and accumulation in winter wheat, especially in wheat plants, have been less studied. Therefore, this paper aimed to study the effects of rhizosphere pH on the growth and development of winter wheat seedlings and NO_3_^−^ ion absorption by roots, as well as the relationship between nitrogen metabolism and growth characteristics. It is hoped that the results of this study will be helpful for breeding wheat varieties resistant to extreme pH conditions, expanding the planting area of winter wheat and increasing the total yield of wheat.

## Materials and methods

### Plant materials and treatment

In this study, two winter wheat varieties widely cultivated in the North China Plain were selected ‘Aikang 58’ (AK58) and ‘Bainong4199’ (BN4199), among which AK58 belonged to dwarf stalk varieties and BN4199 belonged to medium straw varieties, and the biomass of BN4199 was higher than that of AK58. The surface of wheat seeds were disinfected with 1% H2O2 for 24h and then rinsed with distilled water. The pre-soaked seeds were placed in a Petri dish, after which the uniformly sized wheat seedlings were transplanted into the nutrient solution for hydroponic culture, and after growing in the nutrient solution at pH 6.5 for 3 d, the nutrient solution with pH 4.0, pH 6.5 and pH 9.0 was set respectively, and pH 6.5 was used as the control group. Three leaves of uniform-looking wheat seedlings were selected and placed in a nutrient solution containing 5 mM NH_4_NO_3_, 0.75 mM MgSO_4_, 0.5 mM KH_2_PO_4_, 0.072 mM Fe-EDTA, 0.1 mM Na_2_SiO_3_, 50 μM KCl, 10 μM MnSO_4_, 50 μM H_3_BO_3_, 2 μM ZnSO_4_, 1.5 μM CuSO_4_, 0.075 μM (NH_4_)_6_Mo_7_O_24_. The number of seedlings was guaranteed to measure 10–20 plants per indicator, using a completely random design. It was grown in a growth chamber with a photoperiod of 14 h (light intensity of 20000 Lux), a day and night temperature of 22°C/19°C, and a relative humidity of 60%. The incubator was moved randomly every day to reduce position effects. Change the nutrient solution at a fixed time every day. Select 10-day-old (second true leaf folding) wheat seedlings and monitor the response of different rhizosphere pH values to the NO_3_^−^ flux of wheat roots. Plants were treated nitrogen-free for 1 d before NMT (noninvasive microtest technology) assays were used, and seedlings cultured for 2 weeks were selected to measure other indicators.

### Determination of root surface ion flux

In the process of measuring NO_3_^−^ flux, Tris(alkaline component) and HCl(acidic component) were used to adjust the measurement solutions with different pH values (pH = 4.0, pH = 6.5, pH = 9.0). In this experiment, the instantaneous replacement method was adopted (that is, the test solution with different pH values was quickly replaced during the measurement process and the test pH environment of the material was changed) to measure the absorption kinetic characteristics of NO_3_^−^ flux at different acid and base levels. The net NO_3_^−^ flux was measured using NMT (NMT system BIO-IM, LLC, 109 MA, USA). The principle behind this method and the instruments used have been previously described [32–34]. Electrodes, fixers and liquid ion exchange were supplied by Xuyue Technology Co., LTD. (Beijing, China) (http://www.xuyue.net). MageFlux is used for NO_3_^−^ flux data (Younger USA Corp. http://voungerusa.com /mageflux).

To measure H^+^ flux, the pH was adjusted with HCl (acidic component) or Tris (alkaline component) to prepare measuring solutions with different pH values (0.5 mM NH_4_NO_3_, 0.1 mM CaCl_2_, 0.3 mM MES). In this experiment, the instantaneous replacement method was used to measure the absorption kinetics of H^+^ flux at different pH levels (the test solution with different pH values was quickly changed during the measurement process to change the test pH environment of the material). Net H^+^ flux was measured using NMT (BIO-001A, Y ounger USA Sci. & Tech.Co., Amherst, MA, USA). The principle behind this method and the instruments used have been previously described [35, 36]. The electrode, bracket and LIX were provided by Xuyue Technology Co., LTD. (Beijing, China) (http://www.xuyue.net). H^+^ flux data were obtained using MageFlux (Y ounger USA Corp.; http://voungerusa.com/mageflux).

### Root morphological characteristics and dry matter accumulation

Ten plants were selected from each treatment and the root morphology was measured. The collected roots were scanned using an Epson V700 scanner (Seiko Epson Corp, Japan) and analyzed using WinRHIZO 2013 software (Regent Instruments, Cannada Inc). Root length (RL), root surface area (SA), root volume (RV), root diameter (RD) and root tip number (NRT) were measured by root images. Dry matter accumulation in root and aboveground part at different pH values was measured by oven drying method. Dry matter accumulation was measured by drying at 105°C for 30 min and at 70°C for equal weight. The roots were soaked in 20 mM Na_2_-EDTA for 15 min, after which metal ions attached to the root surface need to be removed before drying the roots.

### Nitrogen accumulation

Using the national standard GB2905-1982, the nitrogen content of plant organs was determined by semi-micro Kjeldahl nitrogen determination method.

### NR and GS activities

Nitrate reductase (NR) activity detection using kit (#BC0080) is determined by ultraviolet spectrophotometry (Beijing Solarbio Science & Technology Co., Ltd; http://www.solarbio.com). Ten winter wheat seedlings roots and bud samples were cleaned with distilled water, the surface moisture was absorbed with filter paper, and the seedlings were soaked in the inducer unit for 2 h away from light. The filter paper was used to absorb the water on the surface of the sample, and then frozen at -20°C for 30 min, weighing 0.1 g, then adding 1ml of the extraction solution, and taking 60μL of the supernatant after homogenizing at low temperature. The above operations were carried out in strict accordance with the methods and procedures of the kit instructions. The absorbance was measured at 340 nm with a spectrophotometer, and then the NR activity was calculated based on the fresh weight of the sample.

Glutamine synthetase (GS) activity was determined colorimetrically using a detection kit (#GS-2-Y) (Suzhou Cominbio Biotechnology Co., Ltd., http://www.cominbio.com). Tenwinter wheat seedling roots and stem samples were washed with distilled water and absorbed with surface water, then 0.1 g of the sample was weighed and 1 mL of the extracted assimilation was added, 175 μL of the supernatant was taken, centrifuged at 4°C, 8000 r for 10 min, the reagent was added in strict accordance with the methods and steps dictated by the equipment, then centrifuged at 25°C, 5000 r for 25 min, after which the supernatant was taken after standing at 25°C for 10 min to determine the absorbance at 540 nm.

### Root respiration

The root CO2 exportation rate of wheat seedlings was measured by Li-6400 portable CO2 infrared gas analyzer (IRGA) (Li-COR Inc., Lincoln, NE, USA) at about 2 weeks. A breathing chamber (a rigid PVC pipe with a diameter of 100 mm and a height of 50 mm) was used to randomly select 12 plants and measure root CO2 emission, one group of 4 plants, repeated 3 times. Take wheat roots cultured at different pH, quickly absorb surface moisture, and place on a wet three-layer gauze. The lower edge of the respiratory chamber should be closed as far as possible, and the insertion depth of the respiratory chamber should be set to zero, and then the detection system should be connected to determine the respiratory rate of the root system [37, 38].

### Root activity

Root activity was assessed using the TTC (triphenyl tetrazolium chloride) reduction method. Fifteen seedlings were selected for each treatment, the roots were washed with distilled water, and then the roots were cut to 1-cm-sized cubes and mixed well. Approximately 0.5 g of roots was weighed and placed into a 15-mL centrifuge tube. Each treatment was repeated thrice, 5 mL of phosphoric acid buffer at pH 7.0 and 5 mL of 0.4% TTC were added in succession and kept in the dark at 37°C for 3 h. After incubation, 2 mL of 1 mol/L sulfuric acid were added to stop the reaction. After filtration, 10 mL of methanol were added and incubated at 30°C–40°C for 1–2 h until the root tips were completely white. The OD value was measured at a wavelength of 485 nm using a spectrophotometer.

### Root pH

The ten winter wheat roots were cut off and washed with distilled water, and absorbent paper was used to absorb moisture from the root surface. Two grams of fresh roots were ground, and each treatment was repeated thrice. Approximately 4 mL of distilled water were then added and mixed on an oscillator, left to stand at room temperature for 20 min, centrifuged at 12,000 rpm for 15 min, the supernatant was collected and used in measuring pH with a pH meter.

### Data analysis

SPSS (Statistical Product and Service Solutions) was used to conduct ANOV A assumptions of homogeneity on the displayed data. SigmaPlot 12.0 (Systat Software Inc, Dundas, CA, GER) was used for mapping. The relationship between net ion flux and nitrogen accumulation was analyzed by linear correlation analysis.

## Results

### Characteristics of dry matter accumulation under different treatments

**Fig 1:**
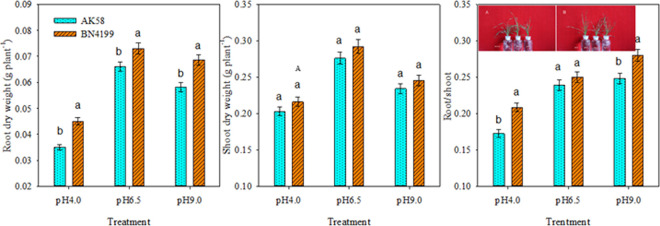
Root dry weight, shoot dry weight and root/shoot of AK58 and BN4199 seedling after 2 weeks treatment at different pH values. Vertical bars indicate the standard deviation, and different letters on the above error bars indicate significant differences among pH values between wheat cultivars at P < 0.05 by the LSD test. As shown in pictures A and B.

Under different rhizosphere pH treatment, there were significant differences in root, aerial biomass and root/shoot ratio of winter wheat (Fig 1). The pH stress did not promote the accumulation of dry matter in both root and aboveground parts. Notably, at pH 6.5, there was a significantly higher accumulation of dry matter in both root and aboveground parts compared to other pH values, with consistent performance observed across the two varieties. Compared with the two varieties, the root dry matter accumulation of ‘BN4199’ was significantly higher than that of ‘AK58’ regardless of acid-base treatment, while the stem dry matter accumulation of ‘BN4199’ under pH 4.0 was significantly higher than that of ‘AK58’, and there was no significant difference between other treatments. Under pH stress, the root/shoot ratio of ‘BN4199’ was significantly higher than that of ‘AK58’. Under pH 6.5 treatment, there was no difference between the two wheat varieties, indicating that ‘BN4199’ had a strong ability to withstand extreme pH.

### Root morphological changes under different pH gradients

As can be seen from Table 1, rhizosphere environment pH had significant effects on root length, root surface area, root volume, root diameter and root tip of winter wheat. Except root diameter, root morphological indexes at different pH values were in the order of pH 6.5 > pH 9.0 > pH 4.0, and root diameter decreased with the increase of pH value, showing the same performance between the two wheat varieties. Compared with the two varieties, the root length, root surface area, root volume and root tip of ‘BN4199’ were significantly higher than those of ‘AK58’.

**Table 1:** Root morphological characteristics of individual plant of wheat under different pH value.

Cultivar	Treatment	Root length (cm)	Root surface area (cm^2^)	Root volume (cm^3^)	Root diameter (cm)	Root tips
AK58	pH4.0	101.91±5.10c	3.25±0.16c	0.066±0.002c	0.040±0.002a	208.13±5.10c
	pH6.5	175.51±8.28a	4.55±0.23a	0.093±0.002a	0.035±0.002b	286.50±8.28a
	pH9.0	156.55±7.83b	3.85±0.19b	0.082±0.002b	0.031±0.002c	252.75±7.83b
BN4199	pH4.0	125.78±5.79c	3.53±0.18c	0.075±0.002c	0.040±0.002a	237.88±5.79c
	pH6.5	192.76±9.64a	4.77±0.24a	0.099±0.002a	0.032±0.002b	367.38±9.64a
	pH9.0	174.25±6.71b	3.96±0.17b	0.085±0.002b	0.031±0.002b	277.88±6.71b

Note: The paper provides two wheat cultivars, Values in the table are means ± SD (n = 10), the root characteristics of same wheat cultivar with different small letters are significantly different at 5% probability level.

### Net flux of nitrate on root surface

**Fig 2:**
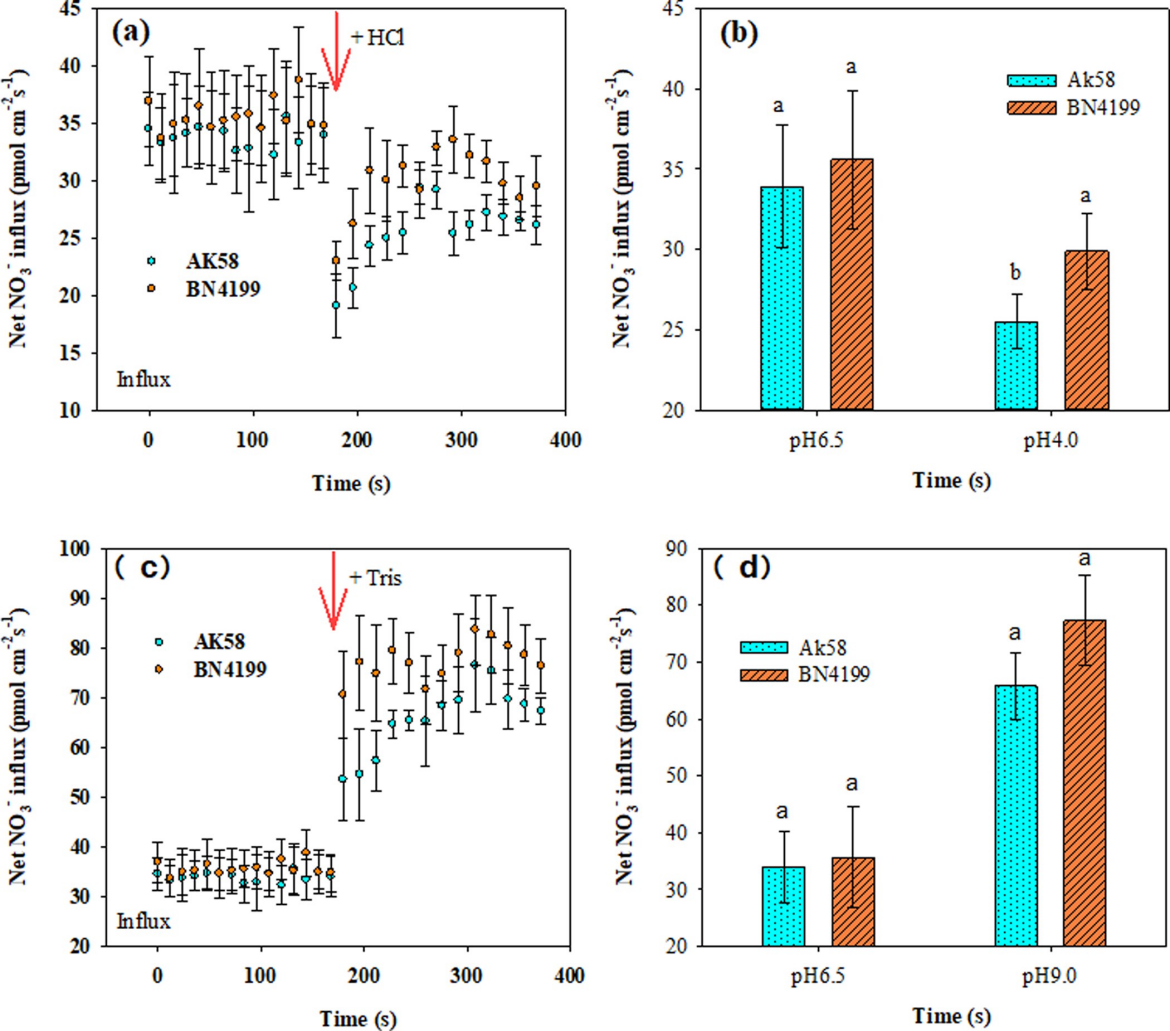
Influence of pH value on NO3- net fluxes on wheat root surfaces. Transient NO_3_^-^ kinetics of different pH value in AK58 and BN4199 roots were measured from root epidermal cells, the dynamic changes in wheat root NO_3_^-^ net fluxes (about 180 s) are presented after changing acidic test liquid (A) or alkaline test liquid (C). The mean ± SE of NO_3_^-^ influxes during the measurement period are shown (n = 6). Acidic or alkaline test liquid was changed at the vertical arrows. B and D were showed mean flux within the measuring period. Vertical bars indicate the standard deviation, and different letters on the above error bars indicate significant differences among pH values between wheat cultivars at P < 0.05 by the LSD test.

Rhizosphere pH affected NO_3_^−^ uptake by wheat seedling roots, and there was a certain difference between ‘AK58’ and ‘BN4199’ (Fig 2). NMT electrode tests showed that the net NO_3_^−^ flux of pH 4.0 treatment was significantly lower than that of other pH treatments, and that of pH 9.0 treatment was significantly higher than that of pH 4.0 and pH 6.5 treatments. The change trend of the two wheat varieties at different pH values was the same. Under pH 6.5 and pH 9.0 conditions, the net NO_3_^−^ flux of ‘AK58’ and ‘BN4199’ was not significantly different, but under pH 4.0 conditions, the net NO_3_^−^ flux of ‘BN4199’ was significantly higher than that of ‘AK58’.

### Determination of nitrogen accumulation in the root system and aerial parts of wheat seedlings

**Fig 3:**
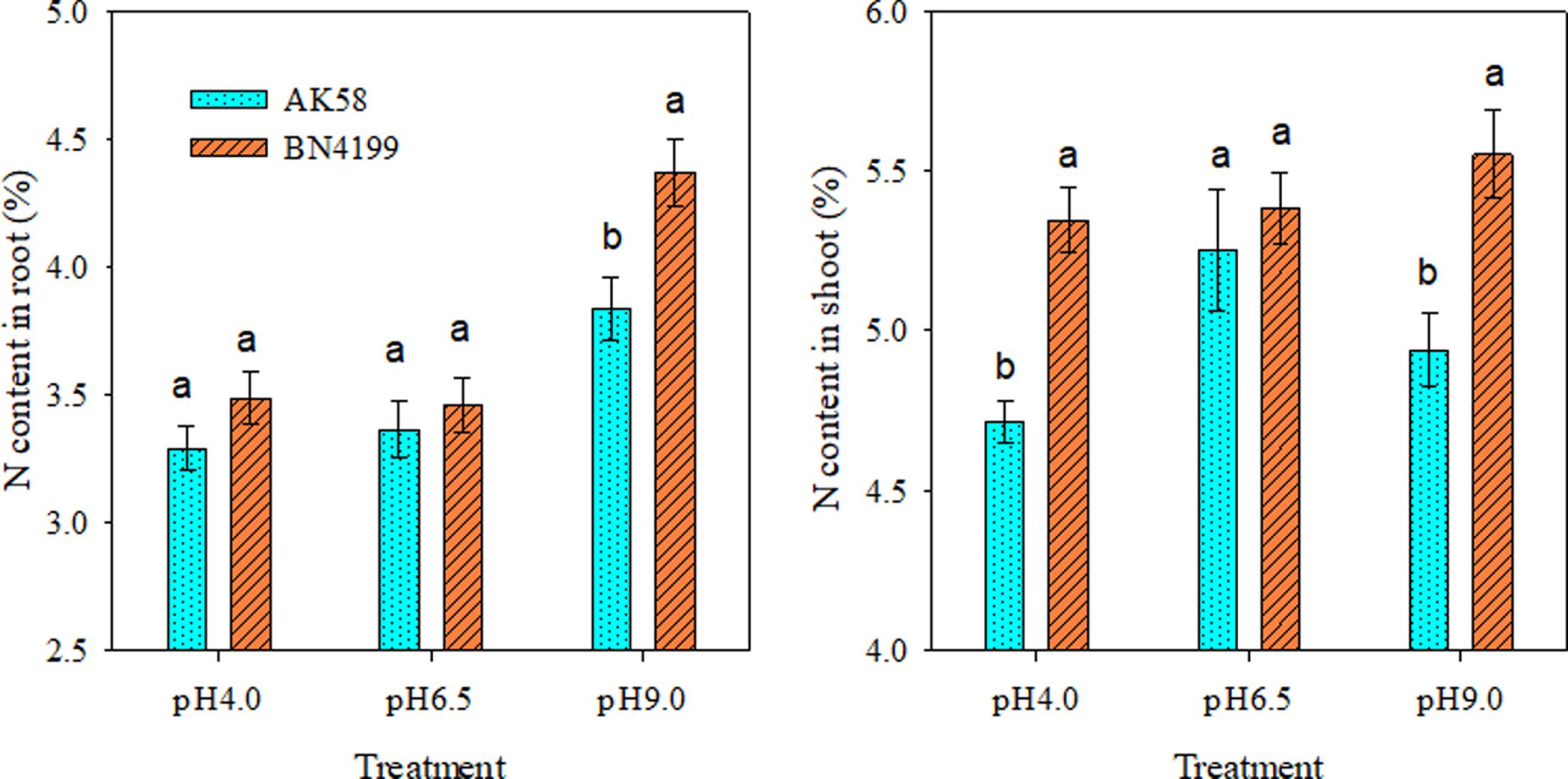
N content in shoot and root of AK58 and BN4199 seedling after 2 weeks treatment at different pH values. Vertical bars indicate the standard deviation, and different letters on the above error bars indicate significant differences among pH values between wheat cultivars at P < 0.05 by the LSD test.

As can be seen from Fig 3, root N content in pH 9.0 treatment was significantly higher than that in other pH treatments, while there was no significant difference in root N content between pH 4.0 and pH 6.5 treatments. At pH 9.0, the root nitrogen content of ‘BN4199’ was higher than that of ‘AK58’. There was no significant difference in the N content in the stem of ‘BN4199’ at different pH values, while the N content in the stem of ‘AK58’ was greatly affected by pH value, and the N content in the stem of ‘AK58’ was successively pH 6.5, pH 9.0 and pH 4.0. Under pH 4.0 and pH 9.0 conditions, the stem nitrogen content of ‘BN4199’ was higher than that of ‘AK58’, but the difference was not significant under pH 6.5 conditions.

### Enzymatic activity assay of root and aerial nitrogen metabolism of wheat seedlings

Wheat nitrogen-metabolizing enzyme activity was shown in Table 2. ‘AK58’ root and stem NR activity did not differ significantly between pH 6.5 and pH 9.0, but was higher than pH 4.0. The root NR activity of ‘BN4199’ was significantly higher than that of other treatments at pH 6.5 and pH 9.0. Root and stem GS activity was highest with pH 6.5, medium with pH 9.0 and lowest with pH 4.0. Compared with ‘AK58’, ‘BN4199’ had significantly higher root and stem NR and GS activities at pH 4.0 and pH 9.0.

**Table 2:** NR and GS activity in wheat plant under different pH value.

Cultivar	Treatment	NR activity (μmol NO_2_^-^ g^-1^ FW h^-1^)		GS activity (μmolg^-1^ FW h^-1^)	
		Root	Shoot	Root	Shoot
AK58	pH4.0	6.73±0.63b	7.10±0.13b	27.00±0.98c	42.17±0.64c
	pH6.5	11.31±0.59a	12.04±0.95a	34.36±0.64a	55.56±1.48a
	pH9.0	12.27±0.47a	12.36±0.11a	29.74±0.92b	46.10±2.10b
BN4199	pH4.0	8.31±0.58c	9.06±0.49b	30.07±0.89c	55.04±1.74b
	pH6.5	21.86±0.27a	9.57±0.13b	55.10±1.09a	69.51±2.03a
	pH9.0	16.37±0.78b	15.65±0.27a	45.00±2.20b	68.59±1.62a

Note: The NR and GS represent the nitrate reductase and glutamine synthetase of wheat seedlings, respectively.

The paper provides two wheat cultivars, Values in the table are means ± SD (n = 4), the NR and GS activity of same wheat cultivar with different small letters are significantly different at 5% probability level.

### Root respiration assay at different pH gradients

**Fig 4:**
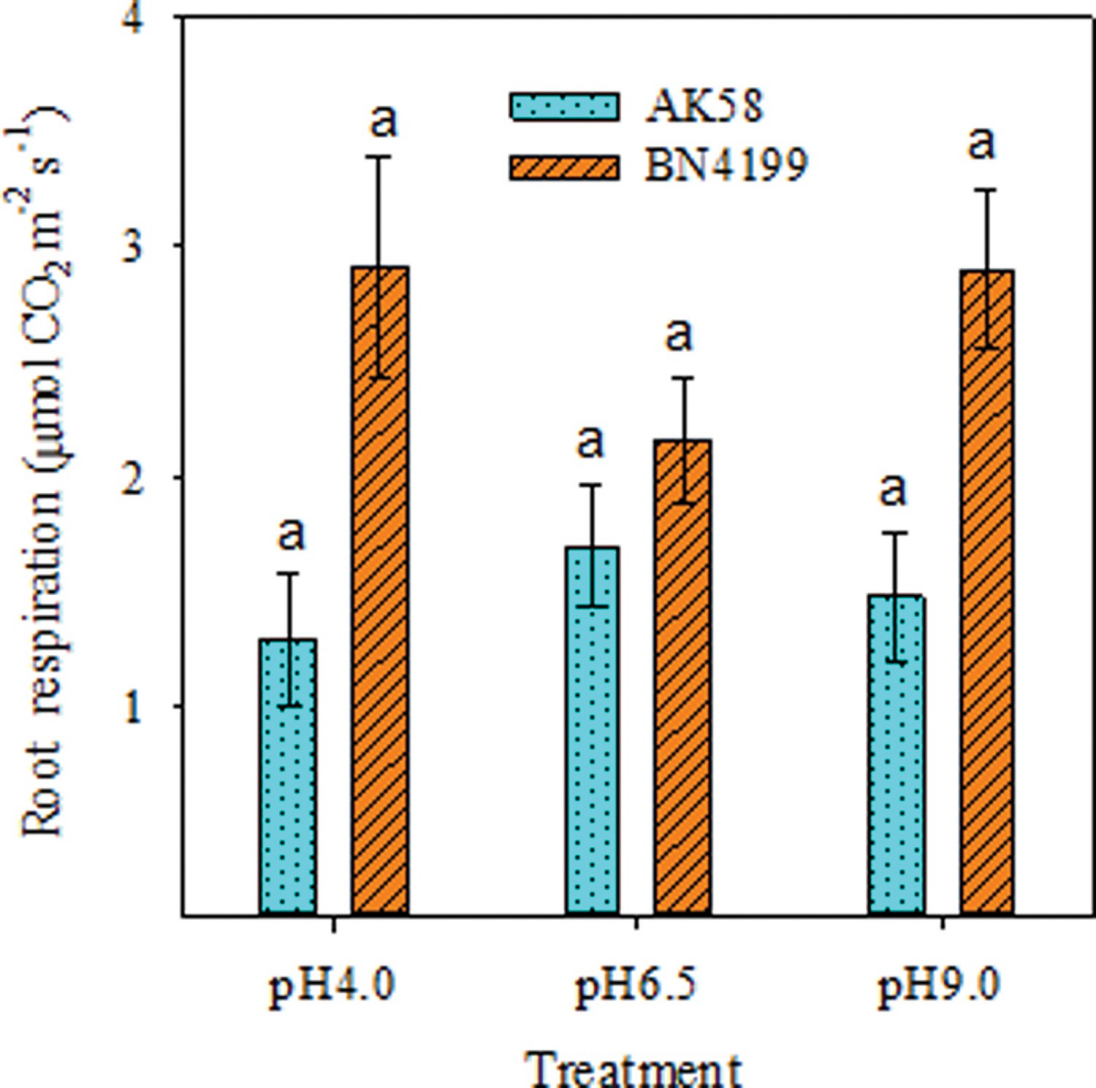
Root respiration of AK58 and BN4199 seedling after 2 weeks treatment at different pH values. Vertical bars indicate the standard deviation, and different letters on the above error bars indicate significant differences among pH values between wheat cultivars at P < 0.05 by the LSD test.

Root respiration of the two wheat varieties at different pH values was shown in Fig 4. The root respiration of ‘BN4199’ did not differ significantly between pH 4.0 and pH 9.0, but was higher than pH 6.5. There was no significant difference in ‘AK58’ root respiration between different pH treatments, but there was a tendency to decrease with the increase or decrease of pH. The root respiration of ‘BN4199’ was significantly higher than that of ‘AK58’ at pH 4.0 and pH 9.0, but the difference was not significant at pH 6.5.

### Net flux of hydrogen ions on root surfaces

**Fig 5:**
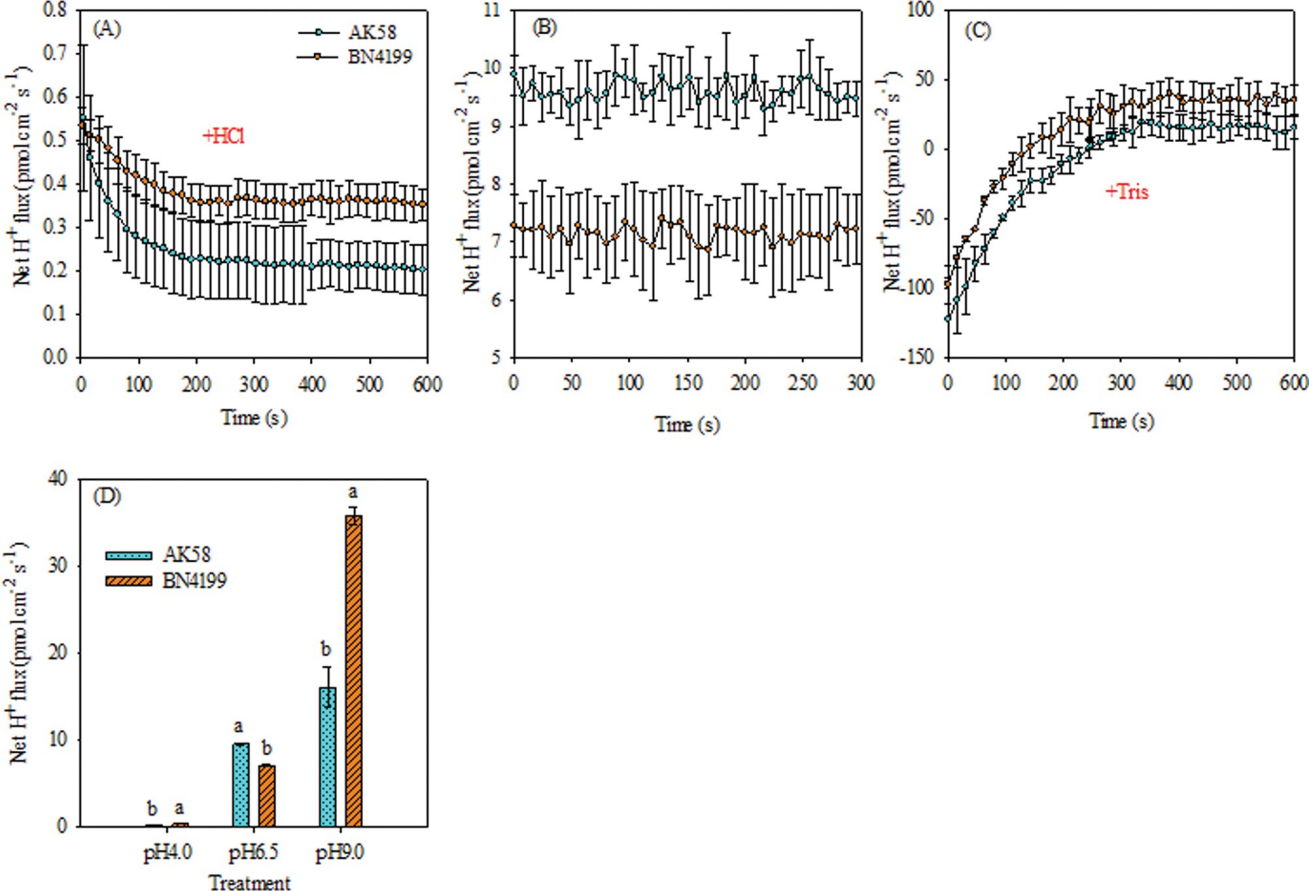
Influence of pH on H^+^ net flux in wheat root surfaces. Transient H^+^ kinetics of different pH levels in AK58 and BN4199 roots were measured in root epidermal cells; the dynamic changes in wheat root H^+^ net fluxes (about600 s) are presented after changing acidic test liquid (A) or alkaline test liquid (C). The mean ± SE of H^+^ net fluxes during the measurement period are shown (n = 5). B shows changes in H^+^ net flux after 5 min at pH 6.5. D shows the mean flux of each treatment 1 min before the end of measurement. Vertical bars indicate the standard deviation, and different letters above the error bars indicate significant differences among pH levels between wheat cultivars at P < 0.05 using the LSD test.

The net H^+^ flux of both varieties showed an outflow when pH 6.5 (Fig 5B). At pH 4.0, the net H^+^ flux of the two varieties decreased significantly compared with the control, while the net H^+^ flux decreased slowly with time, and the net H^+^ outflow rate approached zero (Fig 5A). At pH 9.0, transient performance was characterized by a large net inflow of H^+^ and gradually by a net outflow of H^+^ over time (Fig 5C). The two wheat varieties showed the same trend under different pH levels. The H^+^ outflow rate of ‘BN4199’ was significantly lower than that of ‘AK58’ at pH 6.5, and significantly higher than that of ‘AK58’ at pH 4.0 and 9.0 (Fig 5D).

### Determination of root ability at different pH gradients

**Fig 6:**
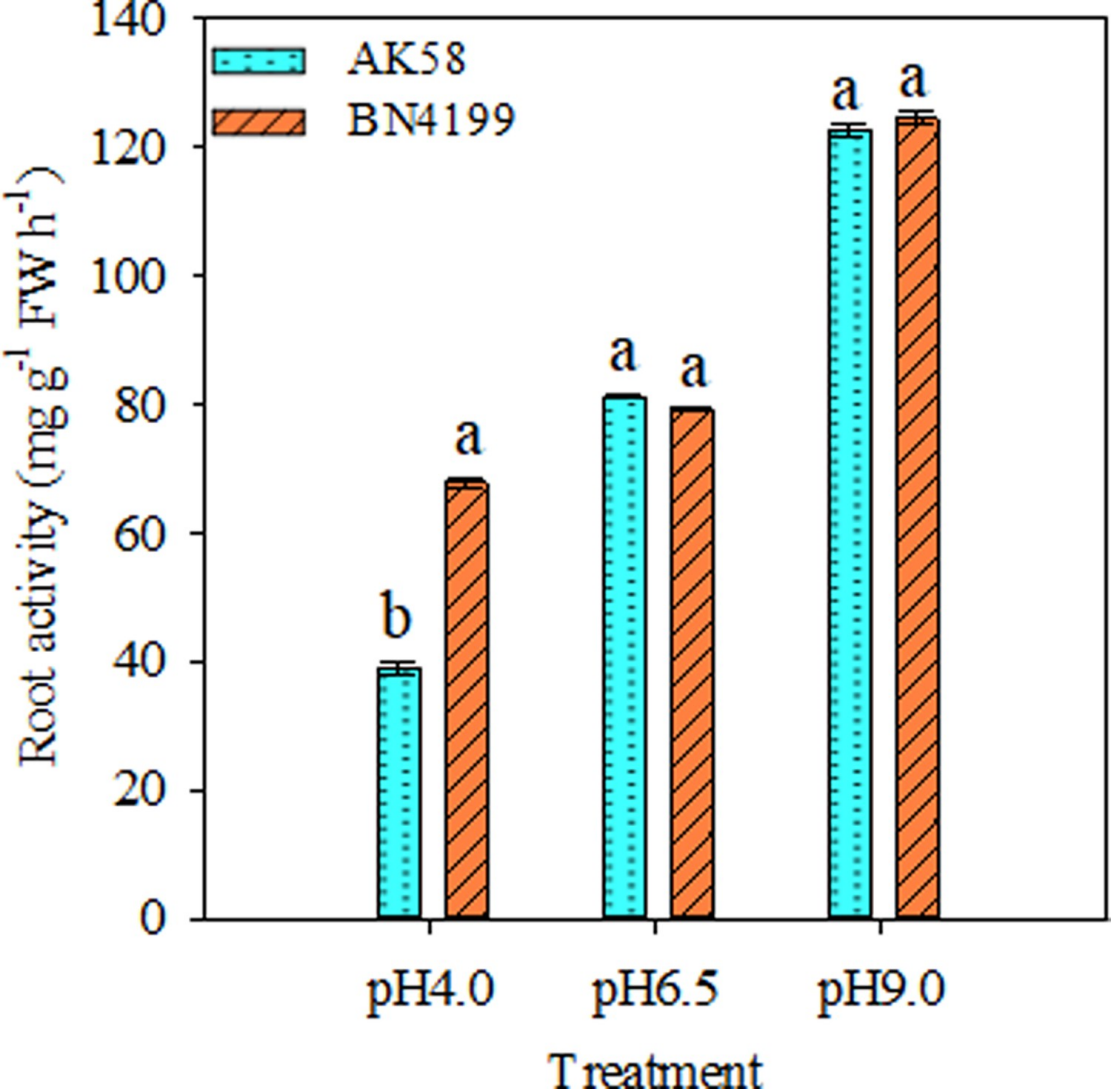
Root activity of AK58 and BN4199 seedlings after 15 d of treatment at different pH levels. Results are shown as means ± SD. Vertical bars indicate the standard deviation, and different letters above the error bars indicate significant differences among pH values between wheat cultivars at P < 0.05 using the LSD.

The root ability of the two wheat cultivars increased with the increase of pH (Fig 6). At pH 6.5 and 9.0, the root ability of the two cultivars was not significantly different, and the root ability of ‘BN4199’ was significantly higher than that of ‘AK58’ at pH 4.0.

### Root pH determination at different pH gradients

**Fig 7:**
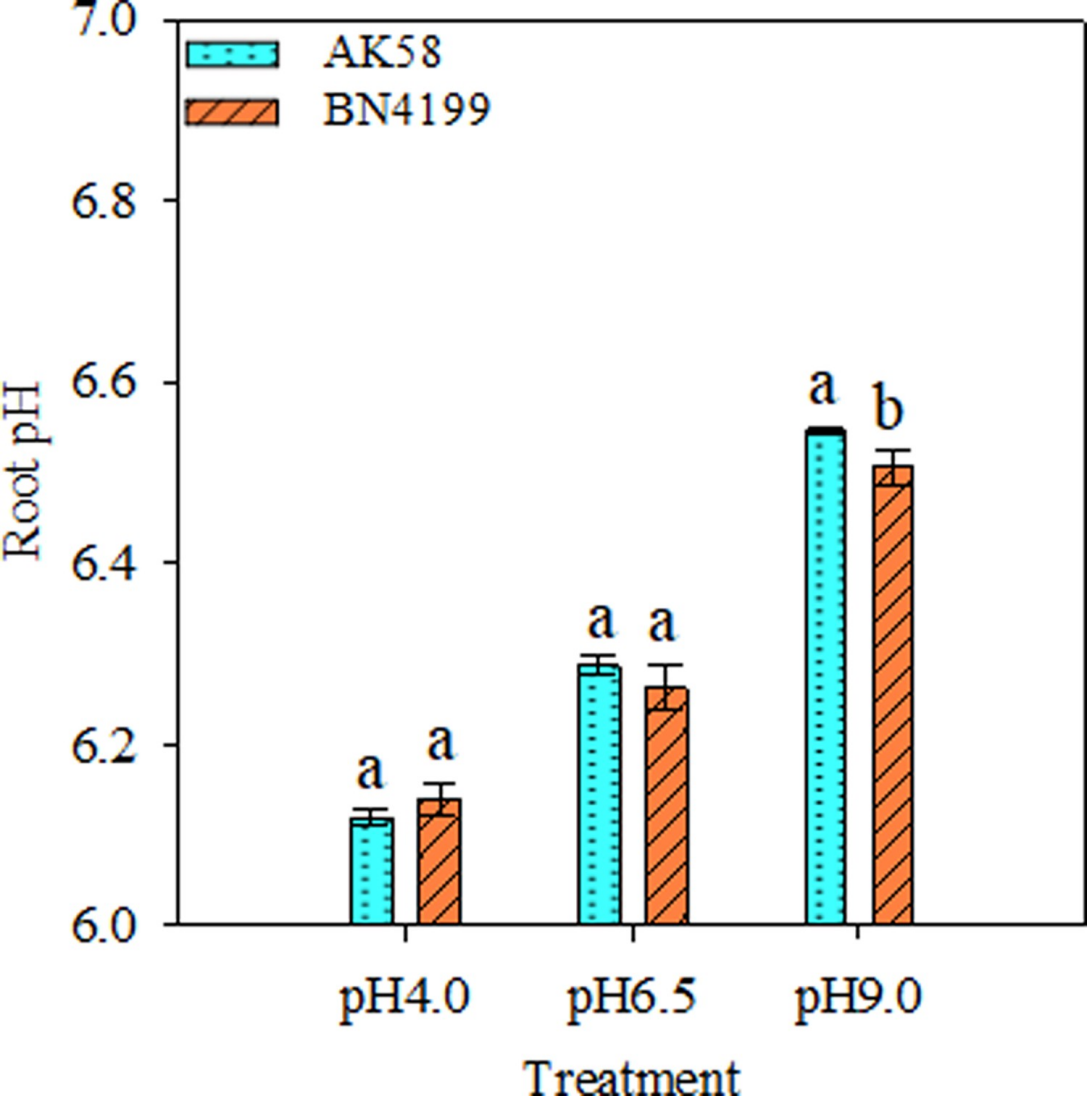
The root pH of AK58 and BN4199 seedlings after 15 d of treatment at different pH levels. Results are shown as means ± SD. Vertical bars indicate the standard deviation, and different letters above the error bars indicate significant differences among pH levels between wheat cultivars at P < 0.05 using the LSD test.

At different pH levels, the root pH values of the two cultivars differed significantly (Fig 7). Under different pH conditions, the root pH values of the two cultivars showed similar trends, and both showed an upward trend of pH 4.0 < pH 6.5 < pH 9.0. The root pH of ‘BN4199’ was lower than that of ‘AK58’ at pH 6.5 and higher than that of ‘AK58’ at pH 4.0, but the difference was not significant. At pH 9.0, the root pH of ‘BN4199’ was significantly lower than that of ‘AK58’. The root pH of ‘BN4199’ and ‘AK58’ decreased by 1.92% and 2.70% at pH 4.0 and increased by 3.99% and 4.15% at pH 9.0, respectively, indicating that the pH variation of ‘BN4199’ was smaller than that of ‘AK58’ under acid-base stress.

### Data analysis

Table 3 showed the correlation between NO_3_^−^ net flux and root, stem weight, root, and stem properties. The net flux of nitrate nitrogen was positively correlated with aboveground NR activity and aboveground N content, and negatively correlated with root diameter. The net flux of nitrate nitrogen was positively correlated with root weight and tip weight, but not significantly. Root weight was positively correlated with tip weight, and root NR activity and root GS activity.

**Table 3:** Correlations between NO3- net flux, root and shoot weight, and root and shoot characteristics (GS, NR, N content, and root traits and vigor, respiration) of winter wheat.

	NR activity in root	NR activity in shoot	GS activity in root	GS activity in shoot	Root length	Root surface area	Root diameter	Root respiration	N content in root	N content in shoot	Root weight	Shoot weight
NO_3_^-^ net flux	0.37	0.89**	0.23	0.30	0.46	0.06	-0.80*	0.27	0.95**	0.33	0.48	0.01
Root weight	0.87*	0.70	0.81*	0.80*	0.99**	0.90**	-0.86*	0.27	0.43	0.70	1	0.87*
Shoot weight	0.85*	0.28	0.83*	0.71	0.89**	0.98**	-0.59	0.06	-0.04	0.52	0.87*	1

The NR and GS represent the nitrate reductase and glutamine synthetase of wheat seedlings, respectively.

* p<0.05

** p<0.01

## Discussion

Soil pH is considered to be an important factor in regulating soil nutrient bioavailability, plant primary productivity, and a range of soil processes including soil microbial community structure and activity, and further affects crop morphology and physiological characteristics [39–42]. In this study, the inhibition of root and dry matter accumulation under high pH conditions was not obvious, resulting in plant dry matter accumulation and root/shoot ratio being greater than that of low pH treatment, indicating that maintaining a high root/shoot ratio at high pH was conducive to the accumulation of total plant biomass (Fig 1). Wang et al. [2] found that soil acidification reduces plant growth by directly inhibiting root growth and function. It can be seen from Fig 1 that low pH conditions have a greater effect on the accumulation of root and stem dry matter in wheat, which proves that low pH conditions have a strong negative effect on wheat growth. It is generally believed that the root system plays an important role in absorbing water, nutrients and providing physical support, and the development status of the root system has an important impact on crop growth and development [43]. Wheat root length, root surface area, root volume, and root tip at extreme pH were significantly lower than pH 6.5, especially at low pH, root elongation and root branching were strongly inhibited (Table 1). The change of pH value significantly changed the morphological characteristics of wheat cultivars [44]. Under different pH gradients, the root morphology of different varieties was significantly different, and the root morphology value of ‘BN4199’ was higher than that of ‘AK58’ at extreme pH and the root/shoot ratio was increased (Fig 1, Table 1). Tang et al. [13] found that genotypic variation in response to extreme pH stress changed the relationship between root morphology and soil conditions due to different sensitivity of root growth to pH changes. Wheat that is resistant to changes in pH conditions adapts to this pH environment by changing the root morphology. Therefore, wheat varieties adapted to different pH conditions can be selected by measuring the root/shoot ratio under hydroponic conditions (Table 1), so as to cultivate a planting environment suitable for different soil properties.

Both the availability of external nutrients and the uptake and utilization of nutrients by the root system can cause changes in the overall morphology of the root system [45, 46]. Giehl and Wiren [46] pointed out that changes in root structure parameters of wheat seedlings reflect different response strategies of plants to available nutrient content. Root structure is regulated by changing the overall lateral root size and then adjusting the uptake and transport of nitrates [47], and hydrogen ion concentration is another important factor affecting plant roots and soil microbial growth. Anion cation exchange in medium, root excretion of organic anions, root respiration, and root-induced redox processes all mediate rhizosphere pH changes. Experiments on the net ion flux of roots of different wheat cultivars showed that the H^+^ and NO_3_^−^ transient efflux rates of wheat roots were significantly higher than those of the control at pH 9.0. At pH 4.0, the root H^+^ and NO_3_^−^ transient efflux rates of wheat were significantly lower than those of the control (Figs 2 and 5), which buffered the extreme pH solution and maintained the ionic balance of the solution to a certain extent (Fig 7), which was conducive to the normal growth of wheat under stress. At the same time, high pH promoted H^+^ net efflux to acidify the rhizosphere, reduce the rhizosphere pH level, and maintain high root ability in wheat, thereby improving the availability of nutrients in the rhizosphere solution, thereby promoting root growth and increasing the root/shoot ratio (Figs 6 and 7). Shen [3] suggests that low pH directly affects the expression of membrane proton pumps in root cells at the transcriptional level, and indirectly affects nutrient uptake by roots and plant growth. H^+^ in low pH environment can disrupt ion absorption patterns and reduce ion transport capacity [48], ultimately leading to decreased root ability, inhibition of root growth, and decrease of root/shoot ratio (Figs 1 and 3). In summary, wheat roots change root pH by releasing or absorbing H^+^, thereby regulating the NO_3_^−^ transport of wheat roots, thereby affecting nutrient availability.

NR and GS are key enzymes in the process of nitrogen metabolism, NR is able to reduce nitrate to nitrite and undergo a series of reactions to generate ammonium, and finally GS converts ammonium to glutamine to participate in the plant growth process [49]. The experiment found that root and aboveground part NR activity was inhibited under low pH conditions, but aboveground part NR activity was higher than other pH treatments at high pH, while GS activity was severely reduced at extreme pH (Table 2). Singh et al. [50]; Zhang et al. [51] found that NO_3_^−^ regulates the transcription, translation, and activation of NR in plants, and the two are positively correlated. The correlation analysis of this study showed that aboveground part NR activity regulated the net uptake of NO_3_^−^ (Table 3), and although high pH conditions increased NO_3_^−^ absorption, the decrease in GS activity led to a decrease in nitrogen assimilation capacity and plant dry matter accumulation (Fig 1, Table 3), so that the root system accumulates a large amount of nitrogen and the nitrogen content increases (Fig 3). Therefore, the main effect of high pH on wheat seedlings was to reduce GS activity, resulting in a decrease in nitrogen assimilation ability, but the effect on root growth was not significant (Fig 3). However, the molecular mechanism of nitrogen uptake and nitrogen assimilation in wheat roots under different pH gradients is unclear and needs to be further studied. The next step is to further investigate how to improve nitrogen use under extreme pH conditions.

Wang et al. [2] reported that the difference in root N ion absorption rate and NR activity between wheat varieties was affected by rhizosphere pH. In this study, the net NO_3_^−^ flux, root morphology indexes, root activity and nitrogen metabolism key enzyme activities of ‘BN4199’ were higher than those of ‘AK58’, indicating that ‘BN4199’ had a strong ability to resist pH changes, but the growth status of both cultivars was weaker than that of other treatments under low pH conditions (Figs 1 and 2). Ejigu et al. [52] argues that wheat is intolerant to soil acidity, consistent with the results of this study. There was a large difference in root development between the two cultivars, but there was no significant difference in stem weight accumulation (Fig 1). This may be due to the fact that wheat is in the seedling stage, and the root system accumulates more nitrogen, which promotes the development of the root system. Under extreme pH conditions, the root respiration rate of ‘BN4199’ was significantly increased (Fig 4), consuming a large amount of organic matter to provide energy for root absorption. At the same time, varieties that are highly adapted to pH environment changes can allocate more dry matter to the root system, promote root development, and achieve a higher root/shoot ratio (Fig 1), laying a solid foundation for the growth of later crops. Because China’s soil shows the distribution characteristics of ’southern acid and northern alkali’ [53, 54], the effect of rhizosphere pH on crops varies among different cultivars, so the response of southern varieties to rhizosphere pH should also be considered in future experiments. The test materials are all northern varieties, which is also the deficiency of this test.

## Conclusion

Wheat has the ability to adapt to high pH, and the tolerance of seedling related traits to different pH in the two wheat cultivars is different under different rhizosphere pH values. Wheat that is tolerant to extreme pH conditions adapts to the pH environment by changing root physiology. The rhizosphere high pH condition enhanced the root activity of wheat by regulating H^+^ efflux, thereby promoting the net uptake of NO_3_^−^ by the roots, increasing the content of N in the roots, and resulting in a higher root/shoot ratio, but the rhizosphere low pH conditions reduced the activity of key enzymes in N metabolism and inhibited the absorption and assimilation of NO_3_^−^ by the roots. The aboveground part of ‘BN4199’ has strong NR activity, which promotes the uptake of NO_3_^−^ in the root, resulting in a high root/shoot ratio. In extreme pH environments, ‘BN4199’ has higher root respiration, providing greater energy for nutrient absorption. The results of this study can be used to screen wheat varieties tolerant to extreme pH conditions at the seedling stage, expand the planting area by screening wheat varieties with high root/shoot ratio and root respiration, and use the above indicators to guide the selection and breeding of new wheat varieties, so as to contribute to the food stability and security of the North China Plain.

## Supporting information

S1 Dataset (XLSX)

## References

[1] Fierer N, Jackson RB. The diversity and biogeography of soil bacterial communities. Proceedings of the National Academy of Sciences. 2006; 103:626–631. doi: 10.1073/pnas.0507535103 16407148 PMC1334650

[2] Wang Y, Yao ZS, Zhan Y, Zheng XH, Zhou MH, Yan GX, et al. Potential benefits of liming to acid soils on climate change mitigation and food security. Global Change Biology. 2021; 27:2807–2821. doi: 10.1111/gcb.15607 33742490

[3] Shen RF. The Behavior of Aluminum in Soil-Plant Systems and Its Adaptive Mechanisms for Plants (in Chinese). Science Press, Beijing. 2008.

[4] Tang CX, Conyers MK, Nuruzzaman M, Poile GJ, Liu D. Biological amelioration of subsoil acidity through managing nitrate uptake by wheat crops. Plant and Soil. 2011; 338:383–397. 10.1007/s11104-010-0552-6.

[5] Jordan-Meille L, Holland JE, McGrath SP, Glendining MJ, Thomas CL, Haefele SM. The grain mineral composition of barley, oat and wheat on soils with pH and soil phosphorus gradients. European Journal of Agronomy. 2021; 126:126281. 10.1016/j.eja.2021.126281.

[6] Feng SW, Gu SB, Zhang HB, Wang D. Root vertical distribution is important to improve water use efficiency and grain yield of wheat. Field Crops Research. 2017; 214:131–141. 10.1016/j.fcr.2017.08.007.

[7] Hinsinger P, Plassard C, Tang C, Jaillard B. Origins of root-mediated pH changes in the rhizosphere and their responses to environmental constraints: A review. Plant and Soil. 2003; 248:43–59. 10.1023/A:1022371130939.

[8] Holland JE, Bennett AE, Newton AC, White PJ, Mckenzie BM, George TS, et al. Liming impacts on soils, crops and biodiversity in the UK: A review. Science of the Total Environment. 2017; 610–611:316–332. doi: 10.1016/j.scitotenv.2017.08.020 28806549

[9] Holland JE, White PJ, Glendining MJ, Goulding KWT, Mcgrath SP. Yield responses of arable crops to liming–An evaluation of relationships between yields and soil pH from a long-term liming experiment. European Journal of Agronomy. 2019; 105:176–188. doi: 10.1016/j.eja.2019.02.016 31007524 PMC6472519

[10] Kost D, Chen L, Guo X, Tian Y, Ladwig K, Dick WA. Effects of flue gas desulfurization and mined gypsums on soil properties and on hay and corn growth in eastern Ohio. Journal of Environment Quality. 2014; 43:312–321. doi: 10.2134/jeq2012.0157 25602565

[11] Zoca SM, Penn C. An important tool with no instruction manual: a review of gypsum use in agriculture. Advances in Agronomy. 2017; 144:1–44. 10.1016/bs.agron.2017.03.001.

[12] Borzouei A, Kafi M, Akbari-Ghogdi E, Mousavi-Shalmani MA. Long term salinity stress in relation to lipid peroxidation, super oxide dismutase activity and proline content of saltsensitive and salt-tolerant wheat cultivars. Chilean Journal of Agricultural Research. 2012; 72:476–482. 10.4067/S0718-58392012000400003.

[13] Tang C, Rengel Z, Diatloff E, Gazey C. Responses of wheat and barley to liming on a sandy soil with subsoil acidity. Field Crops Research. 2003; 80:235–244. 10.1016/S0378-4290(02)00192-2.

[14] Lin XY, Zhang YS, Luo AC, Tao QN. Tolerance of wheat genotypes to Al toxicity in relation to their rhizosphere pH change, NH4+ and NO3- uptake, and nitrate reduction under Al stress. Plant Nutrition and Fertilizer Science. 2002; 8(3):330–334. 10.1006/jfls.2001.0409.

[15] Yang Y, Wang QL, Geng MJ, Guo ZH, Zhao Z. Rhizosphere pH difference regulated by plasma membrane H+-atpase is related to differential Al-tolerance of two wheat cultivars. Plant Soil and Environment. 2011; 57(5):201–206. 10.17221/419/2010-pse.

[16] Dahlgren RA, Saigusa M, Ugolini FC. The nature properties and management of volcanic soils. Advances in Agronomy. 2004; 82:113–182. 10.1016/s0065-2113(03)82003-5.

[17] Seguel A, Cumming JR, Klugh-Stewart K, Cornejo P, Borie F. The role of arbuscular mycorrhizas in decreasing aluminium phytotoxicity in acidic soils: a review. Mycorrhiza. 2013; 23(3):167–183. doi: 10.1007/s00572-013-0479-x 23328806

[18] Guo R, Shi LX, Yang YF. Germination, growth, osmotic adjustment and ionic balance of wheat in response to saline and alkaline stresses. Soil Sci. Plant Nutr. 2009; 55: 667–679. 10.1111/j.1747-0765.2009.00406.x.

[19] Chuamnakthong S, Nampei M, Ueda A. Characterization of Na^+^ exclusion mechanism in rice under saline-alkaline stress conditions. Plant Sci. 2019; 287: 110171. 10.1016/j.plantsci.2019.110171. 31481219

[20] Reid R, Hayes J. Mechanisms and control of nutrient uptake in plants. Int. Rev. Cytol. 2003; 229: 73–114. doi: 10.1016/s0074-7696(03)29003-3 14669955

[21] Liu RR, Cui B, Lu XB, Song J. The positive effect of salinity on nitrate uptake in Suaeda salsa. Plant Physiology and Biochemistry. 2021; 166:958–963. doi: 10.1016/j.plaphy.2021.07.010 34256249

[22] Palmgren MG. Plant plasma membrane H^+^-ATPases: powerhouses for nutrient uptake. Annual Review of Plant Physiology and Plant Molecular Biology. 2001; 52:817–845. 10.1146/annurev.arplant.52.1.817. 11337417

[23] Britto DT, Kronzucker HJ. Nitrogen acquisition, PEP carboxylase, and cellular pH homeostasis: new views on old paradigms. Plant Cell and Environment. 2005; 28:1396–1409. 10.1111/j.1365-3040.2005.01372.x.

[24] Hu XF, Wang D, Ren S, Feng S, Zhang HZ, Zhang JZ, et al. Inhibition of root growth by alkaline salts due to disturbed ion transport and accumulation in Leymus chinensis. Environmental and Experimental Botany. 2022; 200:104907. 10.1016/j.envexpbot.2022.104907.

[25] Masclaux-Daubresse C, Daniel-Vedele F, Dechorgnat J, Chardon F, Gaufichon L, Suzuki A. Nitrogen uptake, assimilation and remobilization in plants: challenges for sustainable and productive agriculture. Annals of Botany. 2010; 105(7):1141–1157. doi: 10.1093/aob/mcq028 20299346 PMC2887065

[26] Vasak F, Cerny J, Buranova S, Kulhanek M, Balik J. Soil pH changes in long-term field experiments with different fertilizing systems. Soil & Water Research. 2015; 10(1):19–23. 10.17221/7/2014-swr.

[27] Gou TY, Yang L, Hu WX, Chen XH, Zhu YX, Guo J, et al. Silicon improves the growth of cucumber under excess nitrate stress by enhancing nitrogen assimilation and chlorophyll synthesis. Plant Physiology and Biochemistry. 2020; 152:53–61. doi: 10.1016/j.plaphy.2020.04.031 32388420

[28] Bahrami H, De Kok LJ, Armstrong R, Fitzgerald GJ, Bourgault M, Henty S, et al. The proportion of nitrate in leaf nitrogen, but not changes in root growth, are associated with decreased grain protein in wheat under elevated [CO_2_]. Journal of Plant Physiology. 2017; 216:44–51. 10.1016/j.jplph.2017.05.011. 28575746

[29] Weligama C, Tang C, Sale PWG, Conyers MK, & Liu D. Localised nitrate and phosphate application enhances root proliferation by wheat and maximises rhizosphere alkalisation in acid subsoil. Plant and Soil. 2008; 312:101–115. 10.1007/s11104-008-9581-9.

[30] Fageria NK, Baligar VC. Growth and nutrient concentrations of common bean, lowland rice, corn, soybean, and wheat at different soil pH on an inceptisol. Journal of Plant Nutrition. 1999; 22(9):1495–1507. 10.1080/01904169909365730.

[31] Tang DD, Liu MY, Zhang QF, Shi YZ, Ma LF, Ruan JY. Effects of Nitrogen Form and Root-zone pH on Nutrient Uptake and Concentrations of Organic Anions in Tea Plants (Camellia sinensis). Journal of Tea Science (In Chinese with an English abstract). 2019; 39(2):159–170.

[32] Sun J, Chen SL, Dai SX, Wang RG, Li NY, Shen X, et al. Nacl-induced alternations of cellular and tissue ion fluxes in roots of salt-resistant and salt-sensitive poplar species. Plant Physiology. 2009; 149(2):1141–1153. doi: 10.1104/pp.108.129494 19028881 PMC2633858

[33] Luo J, Qin JJ, He FF, Li H, Liu TX, Polle A, et al. Net fluxes of ammonium and nitrate in association with H+ fluxes in fine roots of Populus popularis. Planta. 2013; 237:919–931. doi: 10.1007/s00425-012-1807-7 23179443

[34] Yang YJ, Xiong J, Chen RJ, Fu GF, Chen TT, Tao LX. Excessive nitrate enhances cadmium (Cd) uptake by up-regulating the expression of OsIRT1, in rice (Oryza sativa). Environmental & Experimental Botany. 2016; 122:141–149. 10.1016/j.envexpbot.2015.10.001.

[35] Shabala S. Non-Invasive Microelectrode Ion Flux Measurements In Plant Stress Physiology. Plant Electrophysiology. 2006; 35–71. 10.1007/978-3-540-37843-3_3.

[36] Wu HH, Zhu M, Shabala L, Zhou MX, Shabala S. K^+^ retention in leaf mesophyll, an overlooked component of salinity tolerance mechanism: A case study for barley. Journal of Integrative Plant Biology. 2015; 57:171–185. 10.1111/jipb.12238. 25040138

[37] Ruehr NK, Buchmann N. Soil respiration fluxes in a temperate mixed forest: seasonality and temperature sensitivities differ among microbial and root-rhizosphere respiration. Tree Physiology. 2010; 30(2):165–176. doi: 10.1093/treephys/tpp106 20008837

[38] Zhao G, Liu GC, Zhu WZ. Spatial variations in the stem CO_2_ efflux rate of Abies fabri and the response to temperature in the Gongga Mountains. Acta Ecologica Sinica. 2018; 38(8):2732–2742. 10.5846/stxb201705160912.

[39] Caires EF, Garbuio FJ, Churka S, Barth G, Corrêa JCL. Effects of soil acidity amelioration by surface liming on no-till corn, soybean, and wheat root growth and yield. European Journal of Agronomy. 2008; 28(1):57–64. 10.1016/j.eja.2007.05.002.

[40] Ni SQ, Cui QJ. Effects of simulated acid rain on growth of wheat (Secale cereale L.) in north China. International Symposium on Water Resource and Environmental Protection. 2011; 2288–2291. 10.1109/iswrep.2011.5893723.

[41] Bhuyan MHMB, Hasanuzzaman M, Mahmud JA, Hossain MS, Alam MU, Fujita M. Explicating physiological and biochemical responses of wheat cultivars under acidity stress: insight into the antioxidant defense and glyoxalase systems. Physiology and Molecular Biology of Plants. 2019; 25(4):865–879. doi: 10.1007/s12298-019-00678-0 31402814 PMC6656837

[42] Shu X, Daniell TJ, Hallett PD, Baggs EM, Griffiths BS. Soil pH moderates the resistance and resilience of C and N cycling to transient and persistent stress. Applied Soil Ecology. 2023; 182. 10.1016/j.apsoil.2022.104690.

[43] Kareem SHS, Hawkesford MJ, DeSilva J, Weerasinghe M, Wells DM, Pound MP, et al. Root architecture and leaf photosynthesis traits and associations with nitrogen-use efficiency in landrace-derived lines in wheat. European Journal of Agronomy. 2022; 140. 10.1016/j.eja.2022.126603.

[44] Robin AHK, Matthew C, Uddin MJ, Bayazid KN. Salinity-induced reduction in root surface area and changes in major root and shoot traits at the phytomer level in wheat. Jourmal Experimental Botany. 2016; 67:3719–3729. doi: 10.1093/jxb/erw064 26951370

[45] Gruber B, Giehl R, Friedel S, Wiren N. Plasticity of the arabidopsis root system under nutrient deficiencies. Plant Physiology. 2013; 168:161–179. doi: 10.1104/pp.113.218453 23852440 PMC3762638

[46] Giehl RFH, Wirén NV. Root nutrient foraging. Plant Physiology. 2014; 166:509–517. doi: 10.1104/pp.114.245225 25082891 PMC4213083

[47] Kumar SS, Akanksha T, Kumar MP. External nitrogen and carbon source-mediated response on modulation of root system architecture and nitrate uptake in wheat seedlings. Journal of Plant Growth Regulation. 2019; 38(1):283–297. 10.1007/s00344-018-9840-9.

[48] Mitra R, Yadav P, Usha K, Singh B. Regulatory role of organic acids and phytochelators in influencing the rhizospheric availability of phosphorus and iron and their uptake by plants. Plant Physiology Reports. 2022; 27. https://doi.org/10.1007/ s40502-022-00650-3.

[49] Liu LJ, Weng YN, Fang J, Zhao ZJ, Du ST. Understanding the effect of GO on nitrogen assimilation in wheat through transcriptomics and metabolic process analysis. Chemosphere. 2022; 296:134000. doi: 10.1016/j.chemosphere.2022.134000 35192852

[50] Singh R, Parihar P, Prasad SM. Sulfur and calcium simultaneously regulate photosynthetic performance and nitrogen metabolism status in As-challenged Brassica juncea L. seedlings. Frontiers in Plant Science. 2018; 9:772. doi: 10.3389/fpls.2018.00772 29971072 PMC6018418

[51] Zhang QF, Tang DD, Liu MY, Ruan JY. Integrated analyses of the transcriptome and metabolome of the leaves of albino tea cultivars reveal coordinated regulation of the carbon and nitrogen metabolism. Scientia Horticulturae. 2018; 231:272–281. 10.1016/j.scienta.2017.11.026.

[52] Ejigu W, Selassie YG, Elias E, Molla E. Effect of lime rates and method of application on soil properties of acidic Luvisols and wheat (Triticum aestivum, L.) yields in northwest Ethiopia. Heliyon. 2023; 9. doi: 10.1016/j.heliyon.2023.e13988 36873481 PMC9982607

[53] Zhao YY, Zhang ZY, Li ZH, Yang BH, Li B, Tang XD, et al. Comprehensive study on saline-alkali soil amelioration with sediment of irrigation area in northeast China. Arabian Journal of Chemistry. 2023; 16(4). 10.1016/j.arabjc.2023.104608.

[54] Dele Chen, Wang XX, Carrión VJ, Yin S, Yue ZF, Liao Y, et al. Acidic amelioration of soil amendments improves soil health by impacting rhizosphere microbial assemblies. Soil Biology and Biochemistry. 2022; 167: 108599. 10.1016/j.soilbio.2022.108599.

